# Associations between single nucleotide polymorphisms in multiple candidate genes and body weight in rabbits

**DOI:** 10.14202/vetworld.2017.136-139

**Published:** 2017-01-30

**Authors:** Karim El-Sabrout, Sarah A. Aggag

**Affiliations:** 1 Department of Poultry Production, Faculty of Agriculture, Aflaton St., El-Shatby, P.O. Box 21545, University of Alexandria, Alexandria, Egypt; 2 Department of Genetics, Faculty of Agriculture, Aflaton St., El-Shatby, P.O. Box 21545, University of Alexandria, Alexandria, Egypt

**Keywords:** associated genes, body weight, prediction, rabbit, single nucleotide polymorphism

## Abstract

**Aim:**

In this study, we examined parts of six growth genes (growth hormone [GH], melanocortin 4 receptor [MC4R], growth hormone receptor [GHR], phosphorglycerate mutase [PGAM], myostatin [MSTN], and fibroblast growth factor [FGF]) as specific primers for two rabbit lines (V-line, Alexandria) using nucleotide sequence analysis, to investigate association between detecting single nucleotide polymorphism (SNP) of these genes and body weight (BW) at market.

**Materials and Methods:**

Each line kits were grouped into high and low weight rabbits to identify DNA markers useful for association studies with high BW. DNA from blood samples of each group was extracted to amplify the six growth genes. SNP technique was used to study the associate polymorphism in the six growth genes and marketing BW (at 63 days) in the two rabbit lines. The purified polymerase chain reaction products were sequenced in those had the highest and lowest BW in each line.

**Results:**

Alignment of sequence data from each group revealed the following SNPs: At nucleotide 23 (A-C) and nucleotide 35 (T-G) in MC4R gene (sense mutation) of Alexandria and V-line high BW. Furthermore, we detected the following SNPs variation between the two lines: A SNP (T-C) at nucleotide 27 was identified by MC4R gene (sense mutation) and another one (A-C) at nucleotide 14 was identified by GHR gene (nonsense mutation) of Alexandria line. The results of individual BW at market (63 days) indicated that Alexandria rabbits had significantly higher BW compared with V-line rabbits. MC4R polymorphism showed significant association with high BW in rabbits.

**Conclusion:**

The results of polymorphism demonstrate the possibility to detect an association between BW in rabbits and the efficiency of the used primers to predict through the genetic specificity using the SNP of MC4R.

## Introduction

Rabbit has its importance as supplier of meat and it has become one of the necessary sources of food in some country. Molecular techniques in rabbit’s field become very important tools for the detection of markers linked to economical traits [1]. In breeding programs, data utility from associate genes has potential to basically improve the accuracy of selection [2].

Little researches have been made to improve rabbit’s genetic potentiality for high body weight (BW) using genotype based on single nucleotide polymorphisms (SNPs) of candidate genes. The candidate gene method is a powerful process for finding specific loci responsible for genetic variation related with productive traits of interest in animal species [3]. Some of the growth genes were investigated previously such as growth hormone (GH) and myostatin (MSTN) genes which indicated important roles in growth and development of animal. These genes can be considered as candidate genes for production traits of rabbits [4]. Moreover, SNP is a potent method for detecting nucleotide sequence mutation in amplified DNA [5].

The present investigation had been carried out to detect the genetic variation within six growth genes (GH, melanocortin 4 receptor [MC4R], GH receptor [GHR], phosphorglycerate mutase [PGAM], MSTN, and fibroblast growth factor [FGF]) in two rabbit lines namely V-line and Alexandria line using nucleotide sequence analysis, to investigate association between detecting SNP of these genes and BW at market which can be used as specific markers in marker assisted selection (MAS) for high BW in rabbits.

## Materials and Methods

### Ethical approval

The approval from the Institutional Animal Ethics Committee to carry out this study was not required as no invasive technique was used.

### Study design

V-line and Alexandria line rabbits have been used from the stocks available at rabbitry of the Poultry Research Center of Alexandria University, Egypt, during season 2015/2016. V-line is a synthetic maternal line originated at the Department of Animal Science, Politecnica University, Valencia, Spain [6]. The Alexandria line is a synthetic paternal line which comes from the crossing of V-line and black baladi [7]. Rabbits were housed in cages and fed commercial pelleted diet (18% protein and 2600 Kcal/kg). The experiment was conducted on 60 does (30 does from each line) and their kits (443 V-line and 371 Alexandria) (mixed sex). Each kit’s line grouped into high (>1500 g) and low (<1500 g) BW to identify DNA markers useful for association studies with high BW. DNA was extracted from whole blood of 60 rabbits (30 high weight and 30 low weight) from each line at 63 days of age (market age) using DNA isolation kit (Zymo Research, USA) following the manufacturer’s protocol and stored at −20°C until used. To generate polymerase chain reaction (PCR) profiles from rabbit DNA, six different growth primers (GH [231 bp - part of the 5´-flanking region, 5´-untranslated region, exon 1 [CDS], and part of intron 1]; MC4R [500 bp - 5´-flanking region]; GHR [263 bp - exon 1]; PGAM [855 bp - 5´ UTR to intron 2]; MSTN [499 bp - part of the 5’-untranslated region, the coding sequence of exon 1 and part of intron 1]; FGF [288 bp - exon 3]) [4,8-12] were investigate from biosearch technologies (USA) (Table-1). The total PCR reaction volume was 25 µl, included 3 µl of genomic DNA of each group, 1 µl of each primer, 15 µl of ×2 thermo multiplex PCR master mix, and 3.5 µl of RNase-free water. PCR program was performed using a thermal cycler (Thermo^®^ Scientific Corporation, EU) included three main steps: Initial denaturation at 95°C for 5 min, followed by 40 cycles, denaturation at 95°C for 30 s; annealing at 45°C for 30 s and lasted by extension at 72°C for 1 min then an extension cycle at 72°C for 8 min. Amplicones were separated on 1.5% agarose gel, stained with ethidium bromide and visualized under UV transilluminator. The determined DNA bands on the agarose gel were processed for data analysis using GelAnalyser software [13]. The amplified DNA fragments of growth genes were digested with *MSP1*. The restriction fragment length polymorphism (RFLP) was carried out on PCR product according to Zhou *et al*. [14]. The purified DNA was sequenced using the automated sequencer by Macrogen Company (South Korea). Sequence analysis and alignment were carried out using the Codoncode Aligner software (http://www.codon-code.com/aligner).

**Table-1:** List of the six PCR primers used in this study and their sequences.

Primer code	Nucleotide sequence (5’-3’)
GH	Forward 5′- GTATAGTGGGATGGGGTTGG -3′
	Reverse 5′- TTACGCTCCCATTCAGAAGC -3′
MC4R	Forward 5′- CAACCCCAGTTACCAGCACT -3′
	Reverse 5′- GCATTGCTGTGCAGTCCATA -3′
GHR	Forward 5′- AATCCACCTTCAACCCTATC -3′
	Reverse 5′- CGGAGACTTCTTACAATGGC -3′
PGAM	Forward 5′- GAATGCTGATTGGCAGTTGGC -3′
	Reverse 5′- CCAGTTGTCTGAAACCCCTGTG -3′
MSTN	Forward 5′- AATTTTGCTTGCCATTACTGA -3′
	Reverse 5′- TCAGCAGAACTGTTGACATACAC -3′
FGF	Forward 5′- CCTATGCCTCAGCAATACATAGAACT -3′
	Reverse 5′- ATCCAAAGCGAAACTTGAGTCTG -3′

GH=Growth hormone, MC4R=Melanocortin 4 receptor, GHR=Growth hormone receptor, PGAM=Phosphorglycerate mutase, MSTN=Myostatin, FGF=Fibroblast growth factor, PCR=Polymerase chain reaction

### Statistical analysis

Data of individual BW (g) at 63 days of age (market age) are expressed as least square means ± standard error of the mean (SEM). Statistics differences between/within the two lines were determined by ANOVA followed by Duncan’s multiple range test using Statistical Package for the Social Sciences (SPSS) statistical program [15]. The effects (association) of genotypes of growth genes on the BW at market were analyzed by the least-squares method as applied in the general linear model procedure of SPSS program [15] according to the following statistical model:

Y_ijkl_ = µ + L_i_ + G_j_ + BW_k_ + e_ijkl_

Where, Y is the dependent variable, µ is the overall mean of observations, L is the fixed line effect, is the fixed genotype effect, BW (individual BW at market) is the covariate, and e is the residual error.

## Results

**Figure-1:**
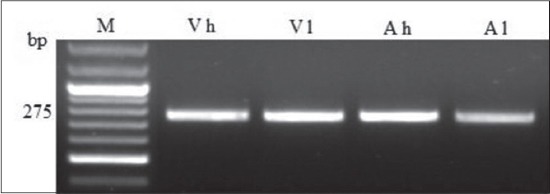
Agarose gel profile of amplified melanocortin 4 receptor gene. M=Marker, Vh=High body weight of V-line rabbits, Vl=Low body weight of V-line rabbits, Ah=High body weight of Alexandria line rabbits, Al=Low body weight of Alexandria line rabbits.

This study aimed to detect genetic polymorphism associated to high BW at market in two rabbit lines named V-line and Alexandria using SNP analysis. We investigated parts of six growth genes (GH, MC4R, GHR, PGAM, MSTN, FGF) for rabbit. All primers were amplified and yielded distinct polymorphic PCR profiles at MW ranged from 200 to 400 bp (Figure-1). RFLP analysis of PCR product using *MSP1* did not produce restriction fragments. The purified PCR products were sequenced in those had the highest and lowest BW in each line and between lines. Alignment of sequence data of 20 rabbits from each group revealed the following SNPs: At nucleotide 23 (A-C) and nucleotide 35 (T-G) in MC4R gene (sense mutation) of Alexandria and V-line high BW. Furthermore, we detected the following SNPs variation between the two lines: A SNP (T-C) at nucleotide 27 was identified by MC4R gene (sense mutation) and another one (A-C) at nucleotide 14 was identified by GHR gene (nonsense mutation) of Alexandria line. Least square means and standard errors (LSM ± SEM) of individual BW at market (63 days) of V-line and Alexandria line rabbits shown in Table-2. The results indicated that Alexandria rabbits had significantly (p≤0.05) higher BW compared with V-line rabbits. MC4R gene showed significant (p<0.05) association with high BW at market between and within the two lines (Table-3). No variations were detected in GH, PGAM, MSTN, and FGF genes. The results of SNP polymorphisms demonstrate the possibility to detect association between BW in rabbits and the efficiency of the used primers to predict through the genetic specificity using the SNP of MC4R.

**Table-2:** Least square means±SEM of individual body weight at market (63 days) in V-line and Alexandria line rabbits.

Lines	BW (g)
V-line (h)	1565.17±21.00^a^
V-line (l)	1322.03±18.10^b^
Alex (h)	1689.54±16.25^a^
Alex (l)	1417.08±20.02^b^
Significance	*

ab Means in the same column with different superscripts are significantly different (p≤0.05). BW=Individual body weight at market (63 days). V-line (h)=High body weight of V-line rabbits, V-line (l)=Low body weight of V-line rabbits, Alex (h)=High body weight of Alexandria line rabbits, Alex (l)=Low body weight of Alexandria line rabbits, SEM: Standard error of mean

**Table-3:** Association between MC4R SNPs and individual body weight at market in rabbit.

SNPs^1^	Trait	Genotypes				p value^2^

		V-line (h)	V-line (l)	Alex (h)	Alex (l)	
MC4R	BW (g)	1565.17±21.00^b^	1322.03±18.10^d^	1689.54±16.25^a^	1417.08±20.02^c^	0.03

Values are presented by the Least square means±SEM.

1 Only significant (sense) association SNPs with BW was listed. BW=Individual body weight at market (63 days). V-line (h)=High body weight of V-line rabbits, V-line (l)=Low body weight of V-line rabbits, Alex (h)=High body weight of Alexandria line rabbits, Alex (l)=Low body weight of Alexandria line rabbits.

2 p value, significance value for multiple comparisons of two lines.

ab Means in the same row with different superscripts are significantly different (p<0.05). SNP=Single nucleotide polymorphism, MC4R=Melanocortin 4 receptor, SEM=Standard error of mean

## Discussion

The use of genotype information as an aid to selection can be a rapid and accurate way to improve the genetic progress in rabbits, especially in the case of high-cost phenotypic recording for many economic traits in rabbit. This study aims to enhance selection efficiency on growth performance of rabbits. We used candidate genes approach to identify DNA markers whose variability could be associated with high BW at market. We examined parts of six growth genes GH, MC4R, GHR, PGAM, MSTN, and FGF for two rabbit lines named Alexandria and V-line. From the nucleotide sequence analysis of the whole amplified fragments and the analysis of the least squares of individual BW at 63 days, we observed a significant association between the MC4R SNPs and high BW of rabbits at market (63 days of age). The two MC4R SNPs detected in high BW rabbits within the two lines may induce the change of the activity or function of MC4R gene in rabbit. These base mutations are sense mutations and lead to transforming amino acids: Valine to leucine and leucine to arginine. These results were in agreement with Fontanesi *et al*. [9] who identified several MC4R polymorphisms associated with finishing weight on other rabbit lines. The scientific principle of this association is based on the fact which indicated that variability within genes coding for protein products involved in key physiological mechanisms and metabolic pathways directly or indirectly involved in determining an economic trait which might probably explain a fraction of the genetic variability for the production trait itself [4].

Moreover, the important role of MC4R gene is focused on the biological regulation which is based on epigenetic mechanisms. The rabbit MC4R gene has been already included on chromosome 9 of the Orycun 2.0 rabbit genome version. It is mainly expressed in the hypothalamus (MC4R), in which plays an area responsible for controlling appetite (feed intake) and satiety, which in turn effect on BW. Mutations in MC4R are the most common genetic cause of reduce gene function and some eliminate function altogether.

In addition, the results declared that all band sequences resulting from the six primers possess 100% identity between the two lines using SNP analysis except MC4R and GHR which indicated differences. Sense mutation of MC4R leaded the change of arginine to lysine. Nonsense mutation of GHR did not result amino acids change. The premier step during the biological process of GH is binding with the GHR, then activating the expression of insulin-like growth factor 1 which influences the growth of animal [16]. The GHR SNP was suggested to associate significantly with carcass traits [10]. Furthermore, no variation (mutation) was detected in GH, PGAM, MSTN, and FGF genes between/within the two lines. Working on GH gene in rabbits, Fontanesi *et al*. [4] found that there are no mutations were identified among the analyzed rabbits. Wu *et al*. [11] observed that no association was found between PGAM and BW at market. Sternstein *et al*. [17] indicated that no SNP variation of MSTN gene was found in the coding region. Othman *et al*. [18] reported a negative association between FGF-5 gene (exon 3) and BW in rabbits. SNPs located at candidate genes underlying economic traits allow prediction of the genetic merit of individuals and combined with guarantee consumer protection. From the analysis of this study, we confirmed that MC4R gene could be a candidate gene to identify genes explaining a fraction of variability of growth performance with potential application in MAS in rabbits.

## Conclusion

According to the results of current research, we found a significant association between MC4R gene polymorphism and high BW at market in rabbits. The results of SNP demonstrate the efficiency of used associated genes to predict through the genetic specificity using the SNPs of MC4R. Moreover, the results are effective in rabbit selection for high BW at market.

## Authors’ Contributions

KE and SAA carried out the experiment design participated in practical work and wrote the manuscript. KE had the primary responsibility for the content of the manuscript. All authors read and approved this manuscript.
